# Treating major depressive disorder with an integrated mind-body intervention: Whole-body hyperthermia and cognitive behavioral therapy, a case report

**DOI:** 10.1016/j.aimed.2024.08.018

**Published:** 2024-08-22

**Authors:** Chelsea J. Siwik, Anoushka Chowdhary, Claudine Anglo, Leena Pandya, Stefanie Roberts, Osnat Lupesko-Persky, Patricia Moran, Wendy Hartogensis, Christopher A. Lowry, Charles L. Raison, Rhonda Patrick, Frederick M. Hecht, Ashley E. Mason

**Affiliations:** a Osher Center for Integrative Health, University of California, San Francisco, CA, United States; b Department of Wellness & Preventative Medicine, Cleveland Clinic, Cleveland, OH, United States; c Department of Psychology, University of Arizona, Tucson, AZ, United States; d Department of Integrative Physiology, University of Colorado Boulder, Boulder, CO, United States; e Department of Psychiatry, School of Medicine and Public Health, University of Wisconsin-Madison, Madison, WI, United States; f FoundMyFitness, San Diego, CA, United States

**Keywords:** Major depressive disorder, Whole-body hyperthermia, Cognitive-behavioral therapy, Integrative health, Case report

## Abstract

**Objectives:**

Major depressive disorder (MDD) is a critical health issue that is inadequately addressed with currently available treatments, indicating a need for novel treatment approaches. This case report describes a patient’s outcomes after completing an integrated mind-body depression intervention that combined cognitive-behavioral therapy (CBT) administered by a clinician with whole-body hyperthermia (WBH) administered using an infrared sauna device.

**Methods:**

The patient, a 37-year-old adult male who identified as agender and Asian, completed eligibility screening and a baseline assessment that included a semi-structured clinical interview and self-report measures of mood and emotional health. The intervention included eight weekly 1-hour CBT sessions and eight weekly WBH sessions. Before each CBT session, the patient completed the Beck Depression Inventory-II (BDI-II). After the patient completed all intervention sessions, we re-assessed the patient’s mood and cognitive and emotional health and collected verbal feedback about their treatment experience.

**Results:**

At baseline, the patient met the criteria for diagnosis of MDD per the Diagnostic and Statistical Manual of Mental Disorders, Fifth Edition, and they were not receiving any form of treatment. From initial contact to the final assessment (14 weeks), the patient evidenced a 25-point reduction on the BDI-II from a score of 28 to 3, reflecting a clinically meaningful decrease from severe depressive symptoms to remission. At the final assessment, the patient no longer met the criteria for MDD.

**Conclusions:**

For this patient, this relatively brief integrative depression treatment was both feasible and efficacious. The patient achieved depression remission in fewer than 14 weeks. These results warrant greater exploration into the potential benefits of this integrative depression treatment.

## 1. Introduction

Major depressive disorder (MDD) is a critical public health issue that is inadequately addressed with currently available treatments. Only 30 % of individuals achieved symptom remission following treatment with the anti-depressant citalopram, and although 67 % achieved symptom remission after subsequently progressing through a 4-step pharmacologic approach, most experienced relapse during the follow-up period [1,2]. Limitations to pharmacologic interventions include limited efficacy, delayed onset, and side effects that impair quality of life and promote treatment non-adherence/discontinuation [3,4]. Non-pharmacologic psychotherapies, including cognitive-behavioral therapy (CBT), are efficacious in reducing depressive symptoms for some [5]; however, depression only resolves in about 25 % of people treated with CBT alone [1,6]. Thus, there is a strong need for additional treatment options for MDD.

Novel interventions, such as whole-body hyperthermia (WBH), target novel mechanisms underlying depression, including thermodysregulation [7,8]. WBH involves raising core body temperature via hyperthermic baths [16,17], infrared saunas [18,19], and other modalities. Temperature dysregulation is the most commonly observed circadian abnormality in depression [9–11]. Studies have shown that, compared to healthy controls, persons with affective disorders have dysregulated thermoregulatory processes that manifest in elevated body temperature without corresponding increases in nocturnal sweating [7,12,13]. These elevations in nighttime body temperature normalize upon clinical improvement in MDD [7,11,14]. Thus, these findings suggest that treatments, such as WBH, which may improve thermoregulation, hold promise as a new avenue for depression treatment [15].

Antidepressant effects have been observed following WBH [15], and our group has observed reductions in depression symptoms following a single WBH session [16–18]. Results from our randomized, double-blind sham-controlled trial (*N*=29) showed that persons with depression who received one session of active WBH (achieving a core body temperature of 38.5 °C), relative to those who received one sham WBH session (core temperature remained <37.8 °C), had significantly larger reductions in depression scores six weeks later [18]. However, prior to this study, no trials to date have reported on the effects of an integrative mind-body intervention that combines the *known* benefits of cognitive-behavioral therapy (CBT) with WBH. Indeed, CBT, which has been found to reduce depression principally using cognitive restructuring and behavioral techniques, is considered the gold standard non-pharmacologic approach to treating MDD; it has decades of empirical evidence documenting its effectiveness [19]. Thus, we developed an intervention comprising both intervention modalities, as each targets distinct factors that may underlie depression processes (i.e., cognitive, “top-down,” and interoceptive, “bottom-up,” signaling [15,20]. The primary outcomes publication from this study provides further detail [21]. Here, we describe the effects of the combination of CBT and WBH in one patient with MDD following the CARE (CAse REporting) guidelines [22].

## 2. Patient information

The patient was a 37-year-old adult male who identified as agender and Asian. At baseline, the patient reported being college-educated, single, without children, and unemployed for approximately two years. The patient’s primary complaint was depression. The patient endorsed depressed mood and loss of interest/pleasure in typically enjoyable activities, accompanied by changes in sleep (insomnia and hypersomnia), loss of energy, feelings of worthlessness, and diminished ability to think and concentrate, leading to distress and impairment in important areas of functioning. The patient reported that their father died when they were age 12, and their mother died unexpectedly at age 22. The patient reported maintaining a close relationship with their sibling but otherwise reported limited social support.

The patient reported being otherwise medically healthy and denied any use of prescription medication. The patient was of average height and weight (body mass index of 21.6) and had blood pressure within normal range (76/137 mmHg). The patient reported engaging in psychotherapy for depression a few times throughout their life but said it was not helpful. The patient said the most recent time they received psychotherapy for depression was approximately two years prior, and the duration of treatment lasted for six months. The patient reported that they had never used psychiatric medications and denied a history of psychiatric hospitalization.

## 3. Clinical findings

The patient met criteria for MDD, recurrent, severe (ICD-10-CM code F33.1) and Social Anxiety Disorder (ICD-10-CM code F40.10), as defined by the Diagnostic and Statistical Manual of Mental Disorders (DSM-5) [23]. The patient did not meet criteria for other mental health disorders (described below). The patient endorsed occasional use of alcohol and marijuana and denied use of other substances.

## 4. Timeline

The patient said their depressive episode began approximately three weeks before the baseline assessment, although they had been experiencing episodes of depression intermittently throughout their adult life. The patient reported the onset of their first depressive episode began before age 25 and estimated experiencing more than three depressive episodes in their life, although they were unable to recall the exact age of onset or the total number of depressive episodes.

## 5. Diagnostic assessment

Before the baseline assessment, the patient completed a phone screen and three online self-report assessments [24]. The online assessments included the BDI-II (with the first two administrations excluding item 9, which assesses suicidal ideation [25]) as the eligibility criteria required a score of 21 or greater and no greater than a 30 % decrease in BDI-II scores between the final two assessments before the baseline assessment. We excluded item 9 from this calculation as it was not administered at screening (the study clinician administered item 9 during the baseline visit). Because 26 days passed between completion of the first and second BDI-II assessment (which exceeded the study protocol’s 21-day threshold), the participant completed a third BDI-II assessment online to confirm eligibility (i.e., they did not show a BDI-II score reduction of more than 30 %). During a phone screen, study staff assessed the patient’s medical health and criteria A for MDD (i.e., depressed mood, loss of interest/pleasure). At the baseline assessment, the study clinician administered an abbreviated version of the Structured Clinical Interview for DSM-5 (SCID) Research Version [26] to fully assess for MDD and to rule out other primary clinical diagnoses. The clinician assessed for anxiety, neurodevelopmental, schizophrenia spectrum, bipolar, obsessive-compulsive, trauma- and stressor-related, dissociative, feeding and eating, and substance-related and addictive disorders. No primary differential diagnoses were considered or ruled out, and the clinician did not encounter any notable challenges when diagnosing this patient with MDD.

The patient completed standardized self-report assessments during treatment: (1) BDI-II [25] before baseline, at the baseline assessment, immediately before each CBT session and at the final assessment; (2) Patient-Reported Outcomes Measurement Information System (PROMIS-29, version 2.1 [27]) at the baseline assessment, immediately before each CBT session, and at the final assessment; (3) Cognitive Flexibility Inventory (CFI [28]) at the baseline and final assessment; as well as (4) Automatic Thoughts Questionnaire-Revised (ATQ-R [29]) at the baseline and final assessment.

The BDI-II total score was calculated by summing ratings for all 21 items, with higher scores indicating more severe depressive symptoms (score range: 0–63). The PROMIS-29 comprises seven subscales: Depression, Anxiety, Fatigue, Sleep Disturbance, Ability to Participate in Social Roles, Physical Function, and Pain Interference. The four items in each subscale were summed to obtain raw scores and then converted to T-scores using PROMIS scoring conversion tables [30]. The CFI total score was calculated by summing the ratings for all 20 items after reverse-scoring negatively worded items, with higher scores indicating greater cognitive flexibility (score range: 20–140). The ATQ-R has 30 negative items and ten positive items. The Negative Score is the sum of the 30 negative items, with higher scores reflecting greater frequency of automatic negative thoughts (score range: 30–150). The Positive Score is the sum of the ten positively worded items, with higher scores reflecting a greater frequency of automatic positive thoughts (score range: 10–50). The ATQ-R also encompasses four subscales: Personal Maladjustment and Desire for Change (score range: 5–25); Negative Self-Concepts and Negative Expectations (score range: 7–35); Low Self-Esteem (based on two items; score range: 2–10); and Helplessness (based on two items; score range: 2–10). Besides the Positive score, for all ATQ-R subscales, higher scores indicate greater fusion with depressive thoughts, while the reverse is true for the positive ATQ-R score.

During the final assessment, the study clinician re-assessed the patient for MDD, and the patient provided verbal feedback on their experience with treatment.

## 6. Therapeutic interventions

The patient completed 8 CBT and 8 WBH sessions each week, in person, at a research clinic at a major academic medical center. Including the time allocated for pre- and post-session assessments, treatment lasted 4.5 h each week (3.5 h, on average, per WBH session and 1 h for each CBT session). The patient started with a WBH treatment and then completed CBT sessions 1–2 days after each WBH session except for the 5th session. The 5th WBH and CBT sessions were completed on the same day, separated by a 3-hour break between beginning with the WBH session. We selected eight sessions of each to ensure adequate exposure (a substantial dose). We provided WBH before CBT, as some prior research has suggested that warmer temperatures may be associated with greater prosocial behavior [31,32].

### 6.1. Cognitive behavioral therapy (CBT)

The CBT protocol was adapted from “Cognitive Behavior Therapy: Basics and Beyond” [33] and “A Therapist’s Guide to Brief Cognitive Behavioral Therapy” [34]. The study clinician (a PhD-level clinical psychologist) and the principal investigators (a licensed clinical psychologist and internal medicine physician with behavioral medicine training) designed the treatment protocol. A study clinician with extensive training and experience administering CBT provided the CBT sessions. Table 1 outlines the session-by-session agenda and the out-of-session homework assigned to the patient to complete between sessions. We gave the patient the option to complete CBT sessions in person or virtually via Zoom, and the patient chose to complete all CBT sessions in person.

### 6.2. Whole-body hyperthermia (WBH)

WBH was administered using the Clearlight Sauna Dome. We used the Mindray iPM-9800 to continuously monitor core body temperature using rectal measurement throughout the session. The patient inserted a thermometer into the rectum before starting the WBH session, which remained indwelling until the post-WBH cooldown period ended. The ambient temperatures in the Sauna Dome reached approximately 57.2 °C and WBH sessions ended once the patient reached a core (rectal) body temperature of 38.5 °C, as used in prior WBH treatments for depression [15,16,18], or when the maximum time was reached (110 or 140 min). The maximum duration for WBH sessions was initially restricted to 110 min for the first four sessions but was increased to 140 min for sessions 5 – 8 to allow sufficient time for the patient to reach the target core body temperature.

Two study staff were present with the patient throughout each WBH session. As done in our prior work [16,18], one staff person sat near the patient’s head and applied cool cloths to the patient’s forehead, scalp, and underneath the neck to keep their head and face comfortable. Study staff directed small fans toward the patient’s face to increase air circulation and provided the patient with water containing an electrolyte drink mix throughout the WBH session. A second study staff person monitored the patient’s core body temperature on the Mindray iPM-9800 machine, which provides a temperature reading every 60 s. After reaching a core body temperature of 38.5 °C for two consecutive minutes, the patient began a 30-minute cool-down period. During the cool-down period, study staff covered the patient in warm towels. The patient’s body temperature typically rose during the first several minutes of the cool-down period before falling.

## 7. Follow-up and outcomes

### 7.1. Clinician- and patient-assessed outcomes

**Fig. 1.**
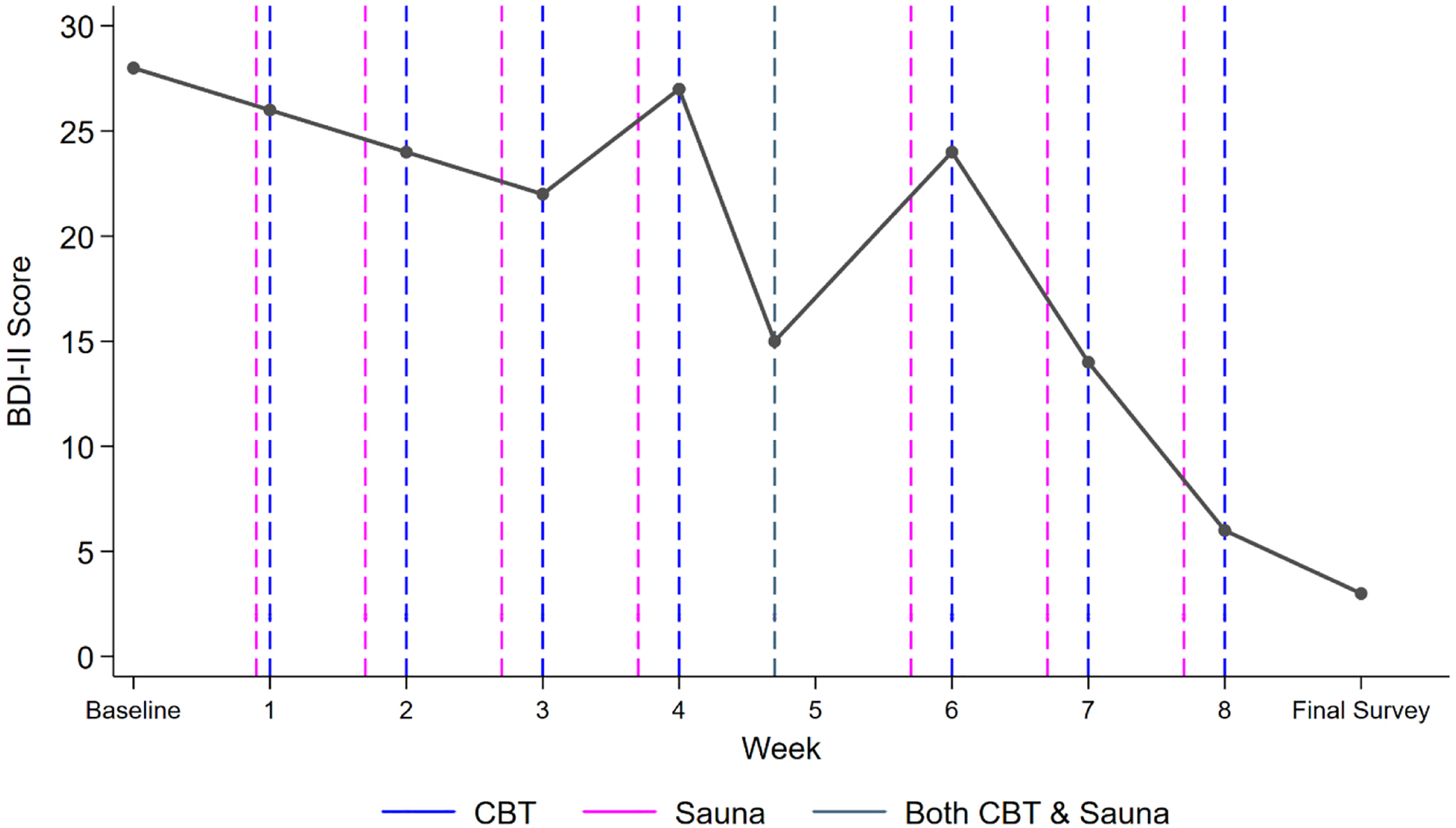
Longitudinal changes in the patient’s depressive symptoms as measured by the BDI-II throughout treatment.

Patient outcomes are summarized in Table 2. As shown in Fig. 1, from the BDI-II administered the day before the baseline assessment to the BDI-II administered at the final assessment (a total of 14 weeks), the patient’s BDI-II score dropped from 28–3, a 25-point reduction, reflecting a clinically meaningful decrease from severe to minimal depression symptoms. The patient also had changes in scores on all PROMIS-29 subscales, reflecting improvement in depression, anxiety (a notable 25-point reduction), fatigue, sleep disturbance, social role function, physical function, and pain interference. The patient also had a 29-point increase on the CFI (cognitive flexibility), suggesting an improved ability to replace maladaptive thoughts with more balanced and adaptive thinking, a core goal of CBT. Lastly, the patient had a 44-point reduction on the Negative Score of the ATQ-R, indicating a sizable reduction in the frequency of negative automatic thoughts, another primary objective of CBT.

At the final assessment, the patient no longer met DSM-5 criteria for MDD. The patient also reported meaningful vocational and social changes in their life, including working for the first time in 2 years and connecting with other people at an exercise club, which was one of the patient’s stated goals during the baseline assessment. The patient also reported being unable to recall a time within the past 2 years during which their symptoms had decreased to such a minimal level of severity.

### 7.2. Intervention adherence and tolerability

The patient attended all CBT and WBH sessions. The patient was engaged and participatory in all CBT sessions and completed all assigned CBT homework. The patient reached the goal temperature of 38.5 °C during 6 of the 8 WBH sessions. The patient did not reach the goal temperature during the first and second WBH sessions when the maximum duration for WBH sessions was restricted to 110 min. During these sessions they reached a maximum temperature of 37.7 °C and 38.4 °C, respectively. The patient consistently met the goal temperature of 38.5 °C for the final 6 WBH sessions, which, on average, entailed 89 min of active heat exposure and ranged from 75 to 110 min. The patient reported consuming alcohol the night before WBH sessions 1, 2, and 6. They otherwise reported adhering to all study instructions (e.g., no marijuana or alcohol consumption 24 h before and after a WBH session). The patient reported all WBH procedures to be acceptable.

### 7.3. Adverse and unanticipated events

The patient reported experiencing mild lightheadedness after 7 of the 8 WBH sessions, a mild headache after 2 of the 8 WBH sessions, and mild tingling in their extremities during 1 WBH session, all of which the patient said resolved by the end of the WBH session or by the end of the day. Study staff advised the patient to drink fluids to alleviate the headache and to move their fingers and toes during subsequent WBH sessions to increase circulation and prevent tingling in the arms and legs. No other adverse or notable unanticipated events occurred during or between WBH or CBT sessions.

## 8. Discussion

For this patient, 8 weekly CBT sessions combined with 8 weekly WBH sessions yielded a clinically significant improvement in depression, as evidenced by a 25-point decrease on the BDI-II, and no longer meeting diagnostic criteria for MDD during the final assessment. In addition, this patient reported other meaningful improvements, including vocational and social changes, such as working for the first time in 2 years. Given that this integrated mind-body treatment approach is highly novel, it remains unclear if CBT, WBH, or a combination of the two drove the observed improvements. Although CBT is well established as an effective non-pharmacologic treatment for depression [5], depression only resolves in about 25 % of persons treated with CBT alone [1,6], and WBH for depression is relatively untested. Prior research has shown that antidepressant responses can follow just a *single* session of WBH within days [16] and that this effect can last as long as six weeks after this single session [18]. This integrated WBH and CBT intervention was substantially longer than prior WBH interventions for depression (i.e., 8 weeks of active treatment and a final assessment more than a week after the final treatment session) [15]. Thus, although future investigation is needed, it is reasonable to postulate that WBH may hold meaningful promise in conferring anti-depressant responses beyond those conferred by CBT alone. Although this treatment was intensive, this patient found the treatment feasible and acceptable. This case report reflects a first step in elucidating the potential antidepressant effects of WBH, highlighting a need for future randomized controlled research with larger samples.

### 8.1. Limitations

Despite these promising results, the current case report has limitations. First, it is unknown how long depression remission persisted for this patient given the lack of long-term follow-up beyond the final assessment, which occurred one week after the final treatment session. MDD is cyclical in nature, so it is possible that the patient’s improvements reflect a natural regression to a baseline state over time. This is unlikely, however, given the other outcomes summarized in Table 2 and functionally meaningful changes the patient reported experiencing for the first time in many years (i.e., returning to work after 2 years of unemployment and an increased ability to engage socially with others). Moreover, although the patient reported experiencing variation in depressive symptom severity for several years before this treatment, the patient reported being unable to recall a time within the past 2 years during which their symptoms had decreased to such a minimal level. Second, the patient was relatively young (37 years of age) and medically healthy (i.e., one comorbid mental health condition and no physical health conditions). It is unknown if this treatment protocol would be feasible or accepted by persons with MDD with more complex health and psychosocial situations given the time-intensive nature of the treatment (i.e., 4.5 h of in-person weekly participation and daily engagement in CBT homework outside of sessions). As this case report involves a single individual, studies with larger numbers and a comparison group are needed to assess the efficacy of this treatment protocol.

## 9. Conclusions

To our knowledge, this case report is the first to describe the first case of depression remission from WBH combined with CBT. It provides preliminary support for using WBH as a safe, tolerable, and novel approach to MDD treatment.

## 10. Patient perspective

During the post-treatment exit interview, the patient reported, “I really liked the sauna portion.” When asked how they would describe their WBH experience to someone considering it, the patient said, “It might be uncomfortable, so be prepared, but the aftereffects are good.” The patient said the frequency of the weekly WBH sessions was acceptable, but they would have preferred CBT more frequently than once per week and recommended against completing a CBT and a WBH session on the same day. The patient said they would complete the same integrated treatment again in the future should they experience a recurrent depressive episode.

## 11. Informed consent

The patient provided informed consent to all procedures and treatments.

## Figures and Tables

**Table 1:** Cognitive behavioral therapy (CBT) session outline.

Session #	Session Content	Homework
1	1. Assess patient’s concerns 2. Create problem list 3. Discuss treatment plan 4. Collaboratively set treatment goals 5. Identify potential barriers	1. Reflect on the session and consider feedback for the therapist 2. Consider revisions to the problem list and treatment plan created during session
2	1. Orient patient to CBT, principles of treatment, and the CBT model 2. Introduce behavioral activation (BA)	1. Complete activity planning and mood monitoring worksheet
3	1. Discuss challenges, barriers, and successes to engaging in BA 2. Increase activity scheduling	1. Complete activity planning and mood monitoring worksheet
4	1. Introduce cognitive side of triangle 2. Explain difference between thoughts and feelings 3. Discuss becoming more aware of automatic thoughts and feelings 4. Begin tracking degree of belief in a thought and intensity of emotion	1. Complete a total of seven 3-column thought records (one per day) prior to the next session 2. Continue engaging in activity planning and mood monitoring
5	1. Cognitive distortions 2. Challenge maladaptive thoughts and beliefs	1. Complete a total of seven 7-column thought records (one per day) prior to next session 2. Continue engaging in activity planning and mood monitoring
6	1. Complete a 7-column thought record 2. Check-in on goals and treatment plan 3. Discuss maintenance of BA. Address stuck points and integrate problem-solving techniques as needed	1. Complete a total of seven 7-column thought record worksheets (one per day) prior to next session 2. Continue engaging in activity planning and mood monitoring
7	1. Continue to work through 7-column thought records 2. Revisit maintenance of BA 3. Discuss any concerns with patient regarding treatment termination next session	1. Complete a total of seven 7-column thought record worksheets (one per day) prior to next session 2. Continue engaging in activity planning and mood monitoring 3. Prepare any questions, concerns, or reflections for next (termination) session
8	1. Review patient’s progress 2. Discuss strategies to maintain improvements 3. Create a relapse prevention plan	

**Table 2:** Changes in the patient’s mood and cognitive and emotional functioning from pre- to post-treatment.

Measure	Assessment Time Point		Total Score Change
	Baseline	Final	
Beck Depression Inventory II (BDI-II)	28	3	−25
Patient-Reported Outcomes Measurement			
Information System–29 (PROMIS–29)			
Subscales			
Depression	66	54	−12
Anxiety	65	40	−25
Fatigue	53	49	−4
Sleep Disturbance	58	51	−7
Ability to Participate in Social Role	41	54	13
Physical Function	44	57	13
Pain Interference	61	52	−9
Cognitive Flexibility Inventory (CFI)	55	84	29
Automatic Thought Questionnaire – Revised			
(ATQ-R) Subscales			
Negative Score	97	53	−44
Positive Score	20	24	4
Personal Maladjustment and Desire for	17	11	−6
Change			
Negative Self-Concepts and Negative	22	12	−10
Expectations			
Low Self-Esteem	4	2	−2
Helplessness	7	5	−2

*Note*. Higher scores on the ATQ-R Negative Score and Positive Score subscales reflect greater frequency of automatic negative thoughts and automatic positive thoughts, respectively.
